# Academy of Medicine

**Published:** 1860-03

**Authors:** John Watson

**Affiliations:** President, in the Chair


*Academy of Medicine.* Regular Meeting, January 18, 1860. Dr. John Watson, *President*, in the Chair.

A paper, on a New Mode of Arresting Surgical Hæmorrhage, by Dr. Simpson, of Edinburgh, was read.

In this paper, the various usual methods of controlling the hæmorrhage arising from surgical wounds were first briefly described, and objections made to them. By the new process of acupressure, Dr. Simpson hoped to overcome, in a great measure, all those difficulties, as by it he expected to arrest the hæmorrhage attendant upon surgical wounds, *without leaving permanently any foreign body whatever* in the wound itself.

Dr. Simpson stated that he had tested the effects of acupressure as a means of effectually closing arteries and stanching hæmorrhage first upon the lower animals, and lately in two or three operations on the human subject. The instruments which he proposed should be used for the purpose were slender needles or pins of passive iron, headed with wax or glass, and in other respects also like the hare-lip needles commonly used by surgeons at the present day, but longer when circumstances require it. They might be coated with silver or zinc on the surface, if such protection were deemed requisite.

At first, Dr. Simpson believed that in using acupressure as a hæmostatic means, it would be necessary to compress the tube of the bleeding artery between two needles, one placed on either side of it. But in his later experiments upon the living as well as the dead body, (as in amputations on the latter, and subsequently injecting tepid water through the arteries, in imitation of the flow of blood,) he had found that the compression of one needle was usually perfectly sufficient to shut up an artery, and that even sometimes, when two or more bleeding points were near, they could be closed simultaneously by the action of one needle or pin. The whole process consists in passing the needle *twice* through the substance of the wound, so as to compress together, and close, by the middle portion of the needle, the tube of the bleeding artery a line or two, or more, on the cardiac side of the bleeding point. The only part of the needle necessarily left exposed on the fresh surface of the wound is the small middle portion of it, which passes over and compresses the arterial tube; and the whole needle is withdrawn on the second or third day, or as soon as the artery is supposed to be adequately closed, thus leaving *nothing* whatever in the shape of a foreign body within the wound or in the tissues composing its sides or flaps. To produce adequate closing pressure upon any arterial tube which it is desired to constrict, the needle must be passed over it so as to compress the tube with sufficient power and force against some resisting body. Such a resisting body will be most frequently found—1st, in the cutaneous walls and component tissues of the wound; 2nd, sometimes in a neighboring bone, against which the artery may be pinned and compressed by the acupressure needle; and 3rd, in a few rare cases it may possibly be found in practice, that a second needle may require to be introduced to serve as a point against which the required compression is to be made. Most commonly the first of these three plans seems perfectly sufficient, and that even in amputation of the thigh. In acting upon this mode, the surgeon may place the tip of the fore-finger of his left hand upon the bleeding mouth of the artery which he intends to compress and close; holding the needle in his right hand, he passes it through the *cutaneous* surface of the flap, and pushes it inward till its point project out to the extent of a few lines on the raw surface of the wound, a little to the right of, and anterior to his finger-tip; he then, by the actions of his right hand upon the head of the needle, turns and directs the needle, so that it makes a bridge as it were

*across* the site of the tube of the bleeding artery immediately in front of the point of the finger with which he is shutting up its orifice; he next, either with this same fore-finger of the left hand, or with the side of the end of the needle itself, compresses the locality of the bleeding arterial orifice and tube, and then pushes on the needle with his right hand so as to make it *re-enter* the surface of the wound a little to the left side of the artery; and lastly, by pressing the needle farther on in this direction, its point re-emerges through the *cutaneous* surface of the flap, and the site of the tube at the bleeding artery is in this way left pinned down in a compressed state by the arc or bridge of steel passed over it. The needle thus passes first from and through the skin of the flap *inward* to the raw surface of the wound, and after bridging over the site of the artery, it passes secondly from the raw surface of the wound *outward* again to and through the skin. Sometimes the needle will be best passed by the aid of the eye alone, and without guiding its course by the finger-tip applied to the bleeding orifice. It compresses not the arterial tube alone, but the structures also placed over and around the *site* of the tube. When the needle is completely adjusted, all of it that is seen on the surface of the raw wound, and that not necessarily so, is the portion of it passing over the site of the artery, while externally, upon the cutaneous surface of the flap, we have remaining exposed more or less of its two extremities—namely, its point and its head. The rest of it is hidden in the structures of the flap or side of the wound. The degree of pressure required to close effectually the tube of an artery is certainly much less than medical practitioners generally imagine; but in the above proceeding the amount of pressure can be regulated and increased, when required, by the acuteness of the angle at which the needle is introduced and again passed out, the cutaneous and other structures of the flap serving as the resisting medium against which the needle compresses the arterial tube. But if it were ever, perchance, necessary to produce greater compression than can be thus accomplished by the needle alone, this increased pressure could be readily obtained by throwing around the two extremities of the needle exposed cutaneously a figure-of-eight ligature, as in hare-lip, with or without a small compress placed between the arc of the ligature and the skin. The process of the adjustment of the needle is difficult to describe shortly by words, but the whole of it is readily seen and imitated when repeated upon a piece of cloth or leather. We fasten the stalk of a flower in the lapelle of our coat by a pin passed exactly in this manner. To compress a bleeding artery against a bone is somewhat more complicated, but not much so. In accomplishing it, we have to introduce from the cutaneous surface a long needle through the flap of the wound obliquely to near the site of the artery, and then compressing, with the fingers of the other hand, or with the end of the needle, the part containing the artery against the bone, we make the needle, after passing over this compressed part, and after testing whether it has closed the vessel or not, enter into the tissues beyond, and if necessary even emerge from, the cutaneous surface on the other side at an angle somewhat oblique to that at which it entered; thus taking advantage of the resiliency and resistance of the soft textures to make them push the needle with the necessary degree of compression against the artery and bone. Arteries in particular parts require special adjustments and modifications to compress them against the neighboring bone, which only experience can point out. There is always sufficient soft tissue on either side of the artery for the needle to get a purchase upon, to compress the arterial tube against the bone or other resistant point. In two cases, Dr. S. had found that branch of the internal mammary artery, which so frequently bleeds in the bottom of the wound after excision of the mamma, easily and perfectly closed by a needle passed through the flap to near the artery, then lifted over it, and (after compressing it so as to stop the flow of blood) pushed onward into the tissues beyond. Possibly, in some amputations, an acupressure needle or needles may yet be passed, immediately before the operation, half an inch or so above the proposed site of the amputation line, so as to shut the principal artery or arteries, and render the operation comparatively bloodless. If so, these needles would serve, at one and the same time, the present uses of both tourniquet and arterial ligatures. Perhaps this will be found, in some cases, a simple and effectual means of compressing and closing the artery leading to an aneurism—as the femoral artery, for example, in popliteal aneurism—changing the operation for that disease into a simple process of acupuncture instead of a process of delicate dissection and deligation, when in any case the milder methods of compression, manipulation, and continuous flexion of the limbs fail. It has been hitherto a difficult problem to obstruct the vessels of the ovarian ligament in ovariotomy, without leaving a foreign body, whether clamp or ligature, upon the stalk of the tumor, to ulcerate and slough through it. If the stalk be transfixed and pinned in its whole breadth to the interior of the relaxed abdominal walls, by one or more acupressure needles, passed through these abdominal walls from without, this difficulty may possibly be overcome. That needles used for the purpose of acupressure, and passed freely through the walls and flaps of wounds, will not be attended by any great degree of disturbance or irritation, is rendered in the highest degree probable by all that we know of the tolerance of living animal tissues to the contact of metallic bodies. Long ago John Hunter pointed out that small shot, needles, pins, &c, when passed into and imbedded in the living body, seldom or never produced any inflammatory action, or none at least beyond the stage of adhesive inflammation, even when lodged for years. Some time ago, when the subject of acupuncture specially attracted the attention of medical men, Cloquet, Pelletan, Poillet, and others, showed that the passage and retention of long acupuncture needles was attended with little or no irritation in the implicated living tissues. The reviewer of their works in the *Edinburgh Medical Journal* for 1827, observes: "It is a *remarkable* circumstance that the acupuncture needles never cause inflammation in their neighborhood. If they are rudely handled or ruffled by the clothes of the patient, they may produce a little irritation; but if they are properly secured and protected, they may be left in the body for an indefinite length of time without causing any of the effects which usually arise on account of the presence of foreign bodies. In one of M. Cloquet's patients, they were left in the temples for eighteen days; and in cases in which needles have been swallowed, they have remained without causing inflammation for a much longer period. It appears probable, from the facts collected on the subject, that metallic bodies of every kind may remain imbedded in the animal tissues without being productive of injury." All the late observations and experiments upon metallic sutures are confirmatory of the same great pathological law of the tolerance of living tissues for the contact of metallic bodies imbedded within their substance. In the operation for hare-lip, surgeons use needles to keep the lips of the wound approximated, often compressing these needles strongly with their figure-of-eight ligatures, and find this measure the most successful means which they can adopt for accomplishing primary adhesion.

The acupressure of arteries, when compared with the ligature of them, appears, as a means of arresting hæmorrhage, to present various important advantages: 1st. It will be found more easy, simple, and expeditious in its application than the ligature. 2d. The needles in acupressure can scarcely be considered as foreign bodies in the wound, and may always be entirely removed in two or three days, or as soon as the artery is considered closed; whilst the ligatures are true foreign bodies, and cannot be removed till they have ulcerated through the tied vessels. 3d. The ligature inevitably produces ulceration, suppuration, and gangrene at each arterial point at which it is applied; whilst the closure of arterial tubes by acupressure is not attended by any such severe consequences. 4th. The chances, therefore, of the union of wounds by the first intention should be greater under the arrestment of surgical hæmorrhage by acupressure, than the ligature. 5th. Pyæmia and surgical fever seem not unfrequently to be excited by the unhealthy suppuration, &c., in wounds which are liable to be set up by the presence and irritation of the ligatures. 6th. These dangerous and fatal complications are less likely to be excited by the employment of acupressure, seeing the presence of a metallic needle has not the tendency to create local suppurations and sloughs in the wound, such as occur at the seats of arterial ligatures. And 7th. Hence, under the use of acupressure, we are entitled to expect both—first, that surgical wounds will heal more kindly and close more speedily; and secondly, that surgical operations and injuries will be less frequently attended than at present by surgical fever and pyæmia.

The discussion on diphtheria was then taken up, and continued through this and the following sitting of the Academy, but nothing new was elicited either as regards the pathology or treatment of the disease.

